# Feeding Intolerance—A Key Factor in the Management of Acute Pancreatitis: A Review

**DOI:** 10.3390/jcm13216361

**Published:** 2024-10-24

**Authors:** Piotr Górski, Agnieszka Swidnicka-Siergiejko

**Affiliations:** Department of Gastroenterology and Internal Medicine, Medical University of Bialystok, ul. M. Skłodowskiej-Curie 24A, 15-276 Białystok, Poland

**Keywords:** acute pancreatitis, feeding intolerance, nutrition, enteral feeding, oral feeding, gastrointestinal injury, predictors, prognosis, severity

## Abstract

Acute pancreatitis (AP) is one of the most common diseases of the gastrointestinal tract, which in 20% of cases can turn into a severe form, with mortality reaching up to 30%. One of the cornerstones of AP treatment is early nutritional treatment. Feeding intolerance (FI) occurs in up to 25% of patients with AP and is associated with a more severe disease course and poorer clinical outcome. Feeding intolerance can have a multifaceted clinical presentation. The early identification of FI risk factors and appropriately conducted nutritional treatment are critical to the course of the disease. In this review, we summarize the current knowledge of feeding intolerance in AP, its pathomechanisms and risk factors, and its impact on disease progression. We also present suggestions for the management of feeding intolerance.

## 1. Introduction

Acute pancreatitis (AP) is a heterogeneous acute inflammation of the pancreas with an often unpredictable course and is one of the most common diseases of the gastrointestinal (GI) tract. The course of AP is usually mild, but in approximately 20% of cases, it can turn into severe acute pancreatitis (SAP), with life-threatening complications requiring advanced medical care in the intensive care unit (ICU). The general mortality rate has been estimated to be around 5%, and it increases in SAP, where it can reach approximately 30% [1]. The incidence of AP is rising globally, and according to recent data, it differs from 3.4 to 73.4 cases per 100,000 worldwide [2]. In the U.S., the hospital admission number per year is estimated at almost 300,000 patients, which generates tremendous hospitalization costs, estimated to be USD 2.6 billion. Progress in our knowledge of the disease and advancements in the management of AP have been associated with a reduction in the mortality rate. However, according to data from 2019, there are still approximately 115,000 AP deaths per year globally and 5000–9000 deaths reported every year in the U.S. [3,4].

The main cornerstones of AP management are early fluid resuscitation, early nutritional treatment, and the maximization of supportive care in the case that a patient develops local or systemic complications. The early identification of SAP risk factors and appropriate interventions play a key role in its management. Taking into account the limited range of resources available to overcome the severe course of the disease, it is important to use available treatments as efficiently as possible [5]. Unfortunately, one of the most common problems in the management of AP is intolerance of nutritional treatment. Feeding intolerance (FI) in AP can develop in patients fed food orally (oral feeding intolerance, OFI) and also fed enterally (enteral feeding intolerance, EFI) via a nasogastric tube (gastric feeding intolerance, GFI) or a nasojejunal tube (nasojejunal feeding intolerance, NJFI). The incidence of FI is estimated at 13% to 16% of orally fed patients [6,7]. The percentage of FI increases with the severity of the disease. In moderate and severe AP courses, EFI reaches 25.8% [8]. FI may contribute to the discontinuation or temporary withholding of therapy and negatively impact the healing process. It is associated with a more severe clinical course, the requirement for ICU management, longer hospitalization, the need for prolonged enteral nutrition (EN), higher morbidity rates, higher costs of medical treatment, and impaired quality of life, and it is an independent predictor of mortality [6,7,9].

The purpose of this article is to summarize the current knowledge of feeding intolerance in acute pancreatitis; its pathomechanisms; risk factors, and impact on disease progression; and methods for improving FI.

## 2. Feeding Intolerance in Acute Pancreatitis

FI is often observed in patients with AP. As mentioned above, it can occur in 13% to 16% of patients fed orally and in up to 26% of patients with a moderate and severe course when fed enterally [6,7,8]. FI is associated with a more severe disease course and a poorer clinical outcome. In an international, multicenter, prospective study that analyzed 1233 patients with AP, 13% of the patients developed OFI. Patients with OFI, compared with patients without OFI, were hospitalized longer (10 vs. 4 days), had higher mortality rates (3% vs. 0.2%), developed pancreatic necrosis more often (29% vs. 13%), and had moderate-to-severe courses of the disease (41% vs. 24%) [6]. It was reported that AP patients who were discharged from the hospital with GI symptoms (vomiting, nausea, diarrhea), poorly tolerated solid diets during their hospitalizations, or had a history of moderate-to-heavy alcohol use had an increased risk of early readmission [10]. In another study, an increased risk of readmission in AP was associated with several factors such as biliary and alcohol etiology of AP, SAP or necrotizing AP, smoldering symptoms or local complications, hepatobiliary complications, and comorbidities. The intolerance of a solid diet was one of the parameters of the risk scores developed to predict readmission in AP [11]. Moreover, patients with FI were found to have an impaired quality of life and use additional healthcare resources [7]. The presence of GI failure together with longer hospitalization in the ICU were found to be independent predictors of mortality [9]. Another study found that GI tract dysfunction may lead to regurgitation and vomiting of gastric contents, which can lead to aspiration and ventilator-associated pneumonia [12].

## 3. Feeding Intolerance Pathophysiology

The pathophysiology of AP as well as FI in AP is complex. It is influenced by many factors that ultimately lead to the development of acute GI injury, i.e., the malfunctioning of the GI tract caused by acute illness [13]. In AP, the acute inflammatory response is followed by an increase in cytokines and (a) systemic inflammatory response syndrome (SIRS), which is connected with early organ damage. In the second phase of the disease (usually after the first or second week), the risk of bacterial infections increases because of the displacement of gut microflora through the damaged intestinal barrier [14]. In a review by Venkatesh K et al., the authors analyzed 23,206 participants in terms of the immunopathological mechanisms of AP. They emphasized that many immune mechanisms contribute to disease development, and the pathways are multifactorial. The immune dysregulation is more intense in SAP than in moderate and mild forms of AP. The immune dysfunction comprises an innate immune response, cytokine profile dysregulation, lymphocyte abnormalities, paradoxical immunosuppression, and failure of the intestinal barrier function [15]. Thanks to the tight intestinal barrier, the human body is protected against pathogens and other potentially harmful substances. “Leaky gut” or leaky gut wall syndrome is a result of barrier disruption, which may allow the entry of bacterial metabolites and endotoxins (e.g., lipopolysaccharides—LPS) into the circulation [12,16].

In turn, the pathomechanisms of FI in AP are not well established. An important role is played by the damage to intestinal integrity that can occur even before the development of organ failure symptoms. This suggests that it is a consequence not only of intestinal hypoperfusion but also of other mechanisms presumably driven by numerous mediators of the inflammatory response that are considered to participate in acute GI injury and directly damage the intestinal barrier, leading to its increased permeability [17,18,19,20,21,22,23,24]. In the study mentioned above, patients with AP who experienced OFI were more likely to meet at least two SIRS criteria on admission (49% vs. 35%) and at 48 h (50% vs. 26%) [6].

Moreover, there are similarities between AP and sepsis, where an excessive release of inflammatory mediators also leads to SIRS. In sepsis, gastrointestinal epithelial cells undergo accelerated apoptosis. As a result, the protective mucous layer in the GI tract becomes thinner, and the integrity of the intestinal mucosa and the intestinal defense barrier is compromised. This leads to the development of pathogenic flora, thereby enabling the entry of toxic media into the submucosa and the body’s circulation, leading to multiple organ damage and further impairing the absorption of nutrients and GI function. As a result, patients may experience FI [25].

An important element of FI in AP patients is gastroparesis, defined as delayed gastric emptying of food solids with or without liquids. Unfortunately, there is a lack of data on motor and endocrine disorders in AP [26,27]. Another explanation of FI in AP can be the formation of peripancreatic collections as their presence is associated with mass effects—GI compression, impaired gastric emptying, and gastric outlet obstruction—increasing the risk of gastric FI [28]. In the study of OFI in AP, patients with peripancreatic collections were 3.5 times more likely to develop OFI than patients without them [7]. A similar mechanism may apply to pleural effusion, although changes in mesenteric lymphatic flow are also suspected [29,30].

In addition, some medical treatments may affect GI motility and thus the development of FI. One of the known side effects of opioid analgesics is their effect of reducing GI motility via μ receptors in the gut. Interestingly, Landy M. Wu et al. found that the use of any analgesics (although opioid drugs predominated) in the early phase of AP affected GI motility, as assessed by a significant increase in the total Gastroparesis Cardinal Symptom Index (GSCI). Most surprisingly, it turned out that a combination of opioids and intravenous fluids, compared with opioids and no fluids, significantly increased the total GSCI score independently of severity and other covariates [31].

**Figure 1:**
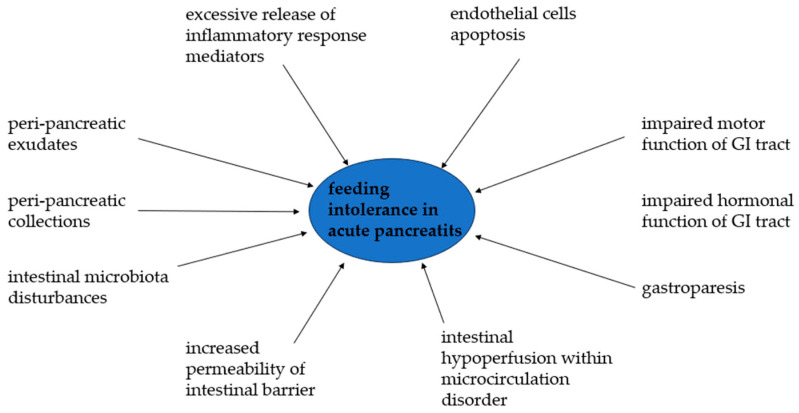
Pathomechanism of feeding intolerance development in acute pancreatitis.

In summary, the pathomechanism of FI development in AP is very complex (Figure 1). However, the aspects are not thoroughly understood. The occurrence of FI is influenced by several factors, such as inflammatory response mediators and their excessive release, endothelial cell apoptosis, impaired motor and hormonal function of the GI tract, gastroparesis, intestinal hypoperfusion with intestinal microcirculation disorder, intestinal barrier damage with an increase in its permeability, and intestinal microbiota disturbances, as well as more tangible factors such as peri-pancreatic collections or exudates [7,26,32,33].

## 4. Feeding Intolerance Risk Factors

The factors related to OFI in AP are not well studied. In a meta-analysis involving more than 2000 patients, Bevan et al. found that patient age, gender, and disease etiology did not affect OFI rates. Interestingly, they found a lower OFI rate in Southeast Asian countries than in Western countries [7]. On the other hand, the most important OFI risk factors in AP were lipase activity more than 2.5× above the upper limit of normal before the initiation of feeding, the presence of peripancreatic collections, pleural effusion, and higher Ranson and Balthazar scores. As for the Ranson Index, its usefulness in the early prediction of OFI was questioned because its full assessment is made 48 h after admission to the hospital. In turn, Balthazar criteria are often inaccurate within the first 48 h, at the time when feeding should be initiated [7]. In turn, a prospective study involving more than 1200 patients with AP concluded that the presence of SIRS 48 h after hospital admission and non-biliary etiology were independent risk factors for OFI. Younger male patients were also more likely to develop OFI [6]. Other OFI risk factors were nicotine and alcohol abuse, which are often associated with a more severe clinical course of AP [34,35]. Moreover, patients who developed OFI were more likely to have higher blood urea nitrogen and hematocrit levels and respiratory failure on admission. Interestingly, the timing of the inclusion of an oral diet did not affect intolerance symptoms [6].

To the best of our knowledge, the risk factors of EFI in moderately severe to severe AP are also not well established. Organ failure, especially respiratory failure, pancreatic necrosis, and peripancreatic fluid collections are risk factors for gastric FI [36,37]. Furthermore, in a retrospective study of 568 patients with moderately severe AP receiving enteral feeding, FI was observed in 32% of patients and was associated with hypertriglyceridemia (odds ratio of 8.13; 95% CI of 5.21–10.07), acute gastrointestinal injury-III status (odds ratio of 5.51; 95% CI of 2.30–7.33), SIRS presence (odds ratio of 6.58; 95% CI of 3.03–8.34), time to start enteral feeding (odds ratio of 7.21; 95% CI of 2.16–9.77), and pancreatic infection (odds ratio of 6.15; 95% CI of 4.94–8.75). Hypertriglyceridemia is often connected with disturbances in the metabolism of glucose and lipids, and uncontrolled hypertriglyceridemia may increase the infection risk and GI tract dysmotility that further leads to FI [38]. The possible risk factors of FI in AP are listed in Table 1.

## 5. Methods Used to Diagnose Feeding Intolerance

Feeding intolerance can have a multifaceted clinical presentation including abdominal pain, nausea, vomiting, bloating, postprandial fullness, early satiety and/or reflux symptoms, inability to advance to an oral diet, worsening of abdominal pain and/or vomiting after resumption of any type of oral diet, or inability to reach the caloric intake target during EN [6,8,26,33].

There are several definitions of FI. Oral feeding intolerance in AP patients may be defined as worsening abdominal pain and/or vomiting after resumption of any type of oral diet [6]. EFI is defined as worsening GI function in response to feeding attempts [39]. In 2012, the European Society of Intensive Care Medicine (ESICM) working group on abdominal problems defined the presence of FI in critically ill patients (nourished by a tube) as when at least 20 kcal/kg BW/day via the enteral route cannot be reached within 72 h since a feeding attempt or if enteral feeding has to be stopped for whatever clinical reason. Unfortunately, this definition, based on a 3-day assessment, cannot be used in the early and rapid assessment of FI in AP [13]. A different approach was taken by Jianbo Li et al., who, in their meta-analysis, proposed using a more measurable and quicker-to-establish parameter—gastric residual volume (GRV). The authors defined FI as GRV ≥ 250 mL ± 50 mL combined with any other GI symptoms [40]. GRV can be measured periodically by either active aspiration of the gastric content by a syringe or passive by gravity drainage into a reservoir [27].

Another definition for GI dysfunction, as well as for FI syndrome and GI symptoms in ICU patients, was introduced in 2012 by The Working Group on Abdominal Problems of the European Society of Intensive Care Medicine (ESICM). They proposed the following four-grade scale of acute GI injury, which is one of the few useful tools to assess the degree of intestinal injury in ICU settings:

- Grade 1: Risk of developing GI dysfunction or failure increased; self-limiting condition. Possible symptoms: Nausea, vomiting, bowel sounds not present, and bowel motility reduced.
- Grade 2: GI dysfunction that requires medical intervention to achieve nutrient and fluid requirements. GI problems do not affect the general condition of the patient. Possible symptoms: Gastroparesis with high GRV or reflux, lower GI tract paralysis, diarrhea, intra-abdominal hypertension (IAH), and visible blood in gastric content or stool.
- Grade 3: GI failure that manifests as sustained intolerance to EN and a general condition that does not improve with treatment and results in worsening MODS, FI, GI paralysis, bowel dilatation, and IAH progression.
- Grade 4: The most severe form of GI failure, which is life-threatening. There is acute critical deterioration of the general patient’s condition with distal organ dysfunctions with the presence of bowel ischemia with necrosis, GI bleeding with hemorrhagic shock, Oglivie’s syndrome, and abdominal compartment syndrome requiring decompression [13].

A new Gastrointestinal Dysfunction Score (GIDS) was developed based on the rationale of the previously developed acute GI injury scale. GIDS is additive to the SOFA score in the prediction of 28- and 90-day mortality or can be used as a stand-alone scale. It is a five-grade scale (from 0 to 4) that assesses the following parameters: peristalsis, diarrhea, vomiting, bloating, GI bleeding symptoms, intra-abdominal pressure, GRV, prokinetics use, oral feeding ability, mesenteric ischemia, and abdominal compartment syndrome. Future studies should validate GIDS before it can be recommended for clinical use in critically ill patients [41].

Gastroparesis plays an important role in the development of FI in AP. A useful supporting scale to assess gastroparesis symptoms is the Gastroparesis Cardinal Symptom Index (GCSI), developed in 2003 by D.A. Revicki et al. It consists of the following subscales: nausea/vomiting, postprandial fullness/early satiety, and bloating. A total of nine symptoms are rated on a scale of 0 to 5 (none to very severe). The final score is the average of the three subscales. The higher the score, the more severe the symptoms. In its original conception, the GCSI assessed the severity of symptoms over a prior two-week period [26] (Table 2).

Because of the likelihood of SAP with the development of multiple organ dysfunction syndrome, GI dysfunction, and its association with poorer clinical outcomes, a daily evaluation of GI function and a clinical examination of the abdomen should be performed in critically ill patients. The GCSI scale may also be helpful. The aim of the daily evaluation is to detect GI dysfunction early, reduce the risk of aspiration-related pulmonary complications and abdominal complications, and optimize the administration of EN.

Interestingly, the current American Society for Parenteral and Enteral Nutrition (ASPEN) guidelines suggest not using GRV in routine clinical practice to monitor ICU patients receiving EN. Instead, they recommend assessing the signs and symptoms of EFI. However, if GRV is still used, ASPEN recommends withholding EN for GRV > 500 mL in the absence of other signs of EN intolerance. In addition, the current European Society for Parenteral and Enteral Nutrition (ESPEN) guidelines recommend delaying EN for patients with GRV > 500 mL/6 h [39,42,43,44].

Considering the role of delayed gastric emptying in FI, other potential tests could be used for its diagnosis. Scintigraphy is the gold standard in the assessment of gastroparesis in outpatients, but it is time-consuming and not widely used in daily clinical practice, especially in critically ill patients. In addition, the current usefulness of breath tests to assess gastric emptying in severely ill patients, including patients with AP, is limited because of the lack of data. In a study that evaluated gastric emptying with a stable isotope breath test compared to scintigraphy, participants with a history of pancreatic disease were excluded [45]. According to recent guidelines, the results of breath tests can be influenced by several factors such as test meal composition, physiological parameters, drugs (with anticholinergic properties, smooth muscle relaxants, and opioids), and concomitant disturbances of gastrointestinal motor and secretory function and hepatic and pulmonary function. Moreover, the exclusion of structural and inflammatory diseases of the gastrointestinal tract, such as inflammatory and malignant disorders, is necessary before testing [46]. Although studies described the use of gastric ultrasound to evaluate FI in ICU settings, its role needs further evaluation [47,48,49]. The results of these studies should be interpreted with caution because of certain limitations, e.g., lack of validated protocols and comparison with other methods. In one study, parameters such as the acute GI injury ultrasonography score and gastric antral cross-sectional area were helpful in predicting FI and 28-day mortality in critically ill patients [49].

## 6. Can Biomarkers Predict Feeding Intolerance?

There are few laboratory parameters that are helpful in the assessment of intestinal barrier function, but they are not widely used in daily clinical practice. The three most common biomarkers of intestinal barrier function are diamine oxidase (DAO), D-lactate, and endotoxin (ETX).

DAO is a secretory protein synthesized only in the epithelial cells of the intestinal villi of the human and mammalian upper intestinal mucosa. It is responsible for catabolizing histamine. DAO activity in serum inversely correlates with the intestinal permeability of the small intestine [50]. D-lactate is produced exclusively by intestinal bacteria and can be found in low concentrations in human blood, and elevated serum levels of D-lactate indicate increased intestinal permeability [51]. Endotoxins (ETXs) are large molecules consisting of a lipid and a polysaccharide (lipopolysaccharide—LPS) and are the major component of the Gram-negative bacteria cell wall. They are usually released after the bacterium’s death. The largest reservoir of bacterial endotoxin is our intestinal tract. After the damage to the intestinal mucosal barrier, endotoxins enter the blood circulation. Their levels can be assessed by anti-endotoxin IgM/IgG antibodies using ELISA [52]. Studies have shown that elevated levels of bacterial endotoxins in the blood are associated with increased intestinal permeability, a more severe disease course, and more frequent FI [21,23,53,54].

Moreover, there are a few lesser-known biomarkers that can be potentially used to assess intestinal barrier function, such as angiopoietin 2 (Ang-2), intestinal fatty acid binding protein (I-FABP), and citrulline. Ang-2, a type of glycoprotein, is an angiogenic growth factor that binds to and inhibits Tie-2 receptors on endothelial cells. This leads to destabilization of the endothelium and increased vascular leakage, resulting in an increase in endothelial permeability. In a prospective study involving 170 patients with AP, Q. Huang et al. showed a strong correlation between elevated serum Ang-2 levels and intestinal permeability. Ang-2 levels were markedly elevated in patients with more prominent FI symptoms [31]. In turn, I-FABP is a cytosolic protein specifically localized in the enterocytes of the small and large intestines, while citrulline is mainly produced by enterocytes in the small intestine. Their blood levels may reflect the damage to enterocytes [55,56]. Researchers have shown that IFAB concentration was significantly higher in AP patients and in more severe courses of the disease. However, citrulline levels were lower in AP [57,58]. Presumably, the citrulline level may also be a marker of FI. Studies conducted on neonates with FI symptoms of various etiologies showed that patients with FI had lower plasma citrulline levels [59].

A test that is used to assess intestinal permeability for clinical research use is the lactulose-to-mannitol ratio test (L:M). It involves oral or tube administration of a specified amount of lactulose and mannitol solutions. These sugars are passively absorbed from the intestine, not extensively metabolized, and excreted unabsorbed in urine in proportion to the quantities absorbed. Their concentrations and ratios in excreted urine are then determined [60]. Agarwal S. et al. found that 96 patients with AP had significantly higher baseline intestinal permeability compared with controls [12]. Sivkov O. et al. claimed that gastric water emptying and acetaminophen absorption tests could predict FI during early AP. The first test involves the administration of 200 mL of water into the stomach and volume evaluation after 30 min and 60 min in ultrasonography. The acetaminophen test is based on the assessment of the acetaminophen concentration in the blood 15–30 min after its administration into the stomach. Both tests are not applicable in wider clinical practice [61]. Other methods to assess intestinal permeability include immunofluorescent staining of intestinal sections; intestinal tight junction assessment in electron microscopy; enteral administration of non-digestible markers such as sugars (lactulose–mannitol test), radioisotopes (e.g., ^51^Cr-EDTA) and polyethylene glycols (PEGs) and their evaluation in urine, blood, or organs; creating isolated intestinal loops and injection of labeled bacterial products, markers, or live bacteria; fecal albumin measurement; measurement of translocated microbial pathogen-associated molecular patterns (PAMPs); and culturing translocated live bacteria [62].

Another method to predict FI was developed by I. Pothoulakis et al., who constructed a statistical model consisting of key clinical variables in order to examine the predictability of OFI. Their results suggested that a clinician has a 66% probability of correctly predicting OFI. However, because of its low to modest predictive accuracy, it is unlikely to be adapted in clinical practice [6].

## 7. Current Nutritional Treatment Recommendation in Acute Pancreatitis

It has been suggested for years that feeding stimulates the release of cholecystokinin, which causes the secretion of proteolytic enzymes, leading to autodigestion and, as a result, further damaging the pancreas. These beliefs led to the use of the nil per os strategy for decades in the treatment of AP [63]. Today, the role of early nutrition in AP is emphasized. Studies have shown that the early inclusion of oral and enteral nutrition leads to reduced complications, reduced length of hospitalization, reduced mortality, improved prognosis, and lower hospitalization costs. When included in a timely manner, nutrition contributes to reducing local and generalized inflammatory responses, improves intestinal barrier integrity, stimulates intestinal motility, prevents bacterial overgrowth, increases splanchnic blood flow, and prevents malnutrition, thus preventing microbiota translocation [64,65]. In turn, it is currently known that prolonged starvation or long-term parenteral nutrition alone leads to changes in the intestinal microbiota and atrophy of the intestinal mucosa, which can result in damage to the intestinal barrier, bacterial translocation, sepsis, and multiple organ dysfunction syndrome [64].

According to the guidelines of ESPEN and the American College of Gastroenterology (ACG), oral nutrition for patients with mild disease should be initiated as soon as it is tolerated by the patient, targeted within 48 h after admission [65,66]. This can be evidenced by the presence of peristalsis or no pronounced nausea, vomiting, or obstruction. The introduction of oral feeding should not depend on the serum concentration of lipase in patients with a predicted mild course of AP. The authors of the ESPEN guidelines suggest initial oral feeding with a low-fat, soft oral diet [65] while the ACG Guidelines authors suggest initial oral feeding with a low-fat solid diet [66]. The authors agree that such an approach allows for faster achievement of the expected caloric supply compared with a liquid diet with identical tolerance [65,66]. If an oral diet is tolerated, the ESPEN guidelines recommend expanding to a standard oral diet. Exceptions are patients with gallstone pancreatitis expecting cholecystectomy and patients with hypertriglyceridemia, where a low-fat diet is recommended [65]. In cases of intolerance to an oral diet, it is recommended to start EN with a standard polymeric diet by nasogastric tube within 24–72 h after admission. In moderate AP, if oral feeding is not tolerated or impossible, EN via nasogastric or a nasojejunal tube should be introduced. In predicted SAP, enteral feeding (NG/NJ tube depending on risk factors) should be initiated within 24–72 h from admission. Continuous infusion is preferred over cyclic or bolus administration. A small peptide-based medium-chain TG oil formula may improve tolerance [14,65,66].

Parenteral nutrition is reserved for patients who are intolerant of EN, in whom we are unable to achieve the target caloric supply, or who have contraindications to EN [14]. The advantage of EN over total parenteral nutrition is evident at the molecular level. EN reduces the pro-inflammatory cytokines IL6, IL-1b, and TNF alpha, while it increases the levels of anti-inflammatory IL11 and IL10 [67,68,69]. Compared with parenteral nutrition, EN is associated with a shorter hospital stay and fewer complications.

## 8. Can We Improve Enteral Feeding Intolerance?

### 8.1. Composition of Nutrition

In patients fed orally, a low-fat, soft oral diet is equally tolerated as a clear liquid diet while being more beneficial according to caloric intake. In one study, the introduction of early oral nutrition was associated with a reduced length of hospitalization [70]. Further clinical studies are needed to determine the possibilities of improving oral feeding in AP [65]. As mentioned above, ESPEN focused on the etiology of AP considering diet modifications. In cases of hyperlipidemia, ESPEN suggests specific medical management including a low-fat diet together with anti-hyperlipidemic drugs, insulin and/or heparin, and plasmapheresis [65].

There are different formulas of EN such as standard polymeric, peptide-based (proteins are hydrolyzed into smaller peptides), immune-modulating (with added antioxidants), disease-specific (for acute and chronic kidney disease, hyperglycemia, hepatic disease), and blenderized (commercially or non-commercially prepared). Standard EN formulas contain intact nutrients like maltodextrin, corn syrup, soy protein isolate, or caseinates, and the fat usually comes from safflower, canola, or soybean oil. They have different caloric densities and may contain fiber [71]. In AP, EPSEN also recommends the use of a standard polymeric diet [65]. Recent studies and meta-analyses found that both polymeric and semi-elemental formulas are safe and well tolerated, with no differences in regard to infections or mortality rates [72,73,74]. However, considering the malabsorption risk in certain subgroups of patients with SAP, semi-elemental diets could be taken into account [65]. In these formulas, protein is hydrolyzed into small-chain peptides, which are easier to digest, and the main source of fats is medium-chain triglycerides [71].

The addition of fiber should not be applied for patients with hemodynamic instability and bowel ischemia risk and should not be used routinely to improve GI motility [71]. However, preliminary studies suggest that the addition of fiber to EN in patients with SAP allows for a faster supply of adequate dietary calories while reducing symptoms of FI. In addition, this improves intestinal permeability and GI motility [75]. In a prospective randomized study, soluble fiber was added to EN via a gastrointestinal tube in 24 patients with SAP; the control group consisted of 22 patients receiving standard EN [75]. In that study, the desired caloric supply was achieved after an average of 5 days and 7 days in the group with fiber and in the control group, respectively. The inclusion of fiber significantly reduced the frequency of symptoms of dietary intolerance (25% vs. 59% in the control group), including abdominal bloating and impaired bowel movements. In addition, the inclusion of fiber improved intestinal mucosal barrier properties (reduced blood levels of diaminoxidase, D-lactic acid, and endotoxins) and modulated the secretion of vasoactive intestinal peptides. In another double-blind RCT that included 30 patients with SAP, the group of patients receiving EN and fiber had a shorter duration of hospitalization and nutrition therapy, faster normalization of the APACHE II index and CRP, and a similar ICU hospitalization length. All patients additionally received partial parenteral nutrition [76].

Glutamine, which has been known for years, is also of interest to researchers. It is an amino acid with immunomodulatory and antioxidant properties. Meta-analyses have shown a beneficial effect of intravenous supplementation with glutamine in patients receiving total parenteral nutrition. Reduced mortality and a reduced number of complications were observed. In turn, the beneficial effect of oral glutamine supplementation on infectious complications or mortality was not confirmed despite the improvement in intestinal permeability, as well as the significant reduction in IL-6 and endotoxemia levels [77,78,79]. Glutamine administered intravenously remains the only immunomodulator that should be administered according to the ESPEN guidelines in parenterally fed patients with SAP when EN is not feasible or contraindicated [65]. Moreover, in several randomized trials, the addition of immunomodulatory omega-3 fatty acid to enteral and parenteral nutrition had beneficial effects, including lowering inflammatory parameters and shortening the duration of nutritional treatment and hospitalization [77,80,81,82]. However, the guidelines do not recommend their use [65,66].

Few studies used modified treatments with the aim of improving the clinical outcomes of AP by affecting the intestinal permeability, bacterial translocation, and reduction in endotoxin levels. In a few randomized trials involving small groups of patients, the addition of prebiotic and probiotic combinations to EN (such as Lactobacillus plantarum 299 with oat supplement and four different Lactobacilli preparations with prebiotics—inulin, beta-glucan, resistant starch, pectin) reduced the rates of SIRS, multiple organ dysfunction syndrome, pancreatic necrosis, and the need for surgical intervention [83,84,85]. In a randomized controlled trial involving 70 patients with SAP, the addition of Bifidobacterium to EN was associated with lower levels of pro-inflammatory cytokines, improved GI function, a lower complication rate, and shorter hospitalization times [86]. In contrast, in a multicenter, randomized, double-blind, placebo-controlled trial involving 200 patients with predicted SAP, prophylactic use of multispecies probiotics was associated with increased mortality despite a beneficial effect on bacterial translocation [87,88,89]. Further studies with the addition of multispecies probiotics confirmed their lack of an effect on intestinal permeability and endotoxemia, despite the reduction in endotoxin levels [90,91]. Based on these studies, it can be speculated that supplementation with certain strains of probiotics may produce beneficial effects in patients with AP [77]. In contrast, supplementation with other probiotics (Lactobacillus acidophilus, Lactobacillus casei, Lactobacillus salivarius, Lactococcus lactis, Bifidobacterium bifidum, and Bifidobacterium lactis) may even be associated with increased mortality in SAP [87]. ESPEN guidelines clearly state that probiotics are not recommended in patients with SAP [65].

### 8.2. Nasogastric Versus Nasojejunal Tube

Currently, EN via a nasogastric tube is recommended because of the ease and quickness of insertion, lower cost, and faster ability to start feeding. Comparisons of nasojejunal and nasogastric administration of EN in RCTs and meta-analyses did not find significant differences in relation to feeding tolerance, rates of complications, or mortality. Moreover, feeding via a gastric tube did not increase hospitalization length or refeeding pain recurrence [92,93,94,95]. Approximately 15% of patients may have digestive intolerance (vomiting, pain) due to delayed gastric emptying or gastric outlet syndrome, and for those patients, nasojejunal feeding may be introduced [65,94,96]. The risk of aspiration should be taken into account and a nasojejunal tube should be used in high-risk patients (e.g., those with organ failure—especially respiratory failure or pancreatic necrosis/fluid collections). Another indication for a nasojejunal tube is intolerance to EN via a nasogastric tube (such as pain and vomiting) [33,65]. The placement of jejunal feeding allows for bypassing gastroparesis symptoms, pancreatic edema, and pseudocysts that compress the stomach or duodenum, but this often requires endoscopy, and mechanical clips may be needed to secure its position. It should be noted that patients at high risk of aspiration should be placed in a more upright position [66]. The data on the role of alternative methods of treatment in AP patients with extended nasogastric or nasojejunal EN are limited considering the risk of patients’ discomfort, sinusitis, nasal trauma, aspirations, and malpositioning. Another approach may be considered, such as a gastrostomy tube or gastro-jejunostomy, although there are no data in AP [97,98].

### 8.3. Continuous Versus Cyclic or Bolus EN Administration

In AP, continuous infusion of EN instead of cyclic or bolus EN administration may improve feeding tolerance [66,99]. The continuous administration of EN was associated with a reduction in diarrhea, while bolus administration was connected with higher gastric volume and mesenteric blood flow [100,101].

The ACG guidelines and ASPEN Guidelines do not specify flow rates, while the ESPEN recommendations in AP suggest that in SAP and intra-abdominal pressure higher than 15 mmHg, EN should be initiated via the nasojejunal route starting at a dose of 20 mL/h [65,66,102]. ESPEN’s Clinical Nutrition in the ICU recommendations advise slowing down or withholding the supply of EN or using prokinetic drugs if there are symptoms of gastric feeding intolerance [100].

### 8.4. Prokinetics and Antiemetics

The ESPEN recommendations in ICU emphasize the advantage of erythromycin (usually at dosages of 100–250 mg intravenously three times a day for two to four days) over other prokinetics in improving gastric FI. Alternatively, intravenous metoclopramide or a combination of metoclopramide and erythromycin can be used. It should be noted that the effectiveness of erythromycin or other prokinetics is decreased to one-third after 72 h, and they should be discontinued after three days. In patients with gastric FI not solved with prokinetic agents, enteral feeding via a nasojejunal tube should be used [39]. It should be underlined that both metoclopramide and erythromycin have adverse events including QT prolongation, cardiac toxicity, tachyphylaxis, and bacterial resistance [103]. In addition, the use of metoclopramide is linked to tardive dyskinesia and akathisia, although reported adverse events in critically ill patients are rare [104]. In addition, in a systemic review and meta-analysis of 13 RCTs comparing prokinetics with a placebo, it was found that there is moderate-quality evidence that prokinetics reduce FI in critically ill patients compared with the placebo or no intervention. However, the influence on other clinical outcomes (e.g., pneumonia, mortality, and hospitalization length) was unclear [105]. Metoclopramide and erythromycin as prokinetics must be used with caution with the close monitoring of side effects.

In a recent RCT study, itopride was well tolerated with superior efficacy to metoclopramide in critically ill patients with intolerance of EN. However, because of the methodological limitations of the study, the conclusions must be interpreted with caution, and further research is needed [106]. Other prokinetics such as camicinal (GSK962040), a motilin agonist, and ulimorelin, a ghrelin agonist, were also found to be useful in critically ill patients with FI [107]. Further research on the safety and efficacy of new prokinetics is necessary before their clinical use. To the best of our knowledge, there is a shortage of studies on the use of other prokinetics in AP.

In a recent population-based study with a total of 1030 patients with AP, the administration of the antiemetic ondansetron was associated with better 90-day outcomes [108]. A single-center, randomized study with a total of 80 patients with IAH within two weeks of AP onset showed that patients who received intramuscular neostigmine, in addition to conventional therapy, reduced IAH faster (−18.7% vs. −5.4% in patients with conventional therapy only) and prompted defecation [109]. Further evidence from a larger, placebo-controlled, double-blind trial is needed to evaluate the results.

### 8.5. Specific Situations

- Patients with SAP and necrosectomy.

According to the ESPEN guidelines, in patients undergoing minimally invasive necrosectomy, oral food intake is safe and feasible. Based on the clinical status of patients, it should be started within the first 24 h after the procedure. In patients who are unable to be fed orally, nasojejunal EN is advised. PN is indicated in patients who do not tolerate EN or who are unable to tolerate targeted nutritional requirements or with contraindications for EN [65].

- Patients with SAP and intra-abdominal hypertension.

IAH is a common complication in SAP that may lead to worse GI blood flow, enterogenic infections, and intestinal paralysis, resulting in digestive intolerance. The ESPEN guidelines suggest that in patients with intraabdominal pressure < 15 mmHg, early EN can be initiated through a nasojejunal tube as the preferred route or a nasogastric tube. When the intra-abdominal pressure is more than 15 mmHg, EN can be administered via a nasojejunal tube starting at 20 mL/h with special monitoring of food intolerance symptoms. In the case of intraabdominal pressure > 20 mmHg or abdominal compartment syndrome, we should stop EN and start PN [65].

A summary of the ESPEN and ACG guidelines regarding nutrition in AP and FI is presented in Table 3.

## 9. Conclusions

Currently, predicting feeding intolerance in acute pancreatitis remains a challenge, and the present evaluation system needs to be improved. Feeding intolerance may affect up to 25% of the patients and may have a significant impact on the course of the disease. There is no perfect predictor of feeding intolerance that can be routinely used in clinical practice. However, early recognition of feeding intolerance symptoms and its risk factors may allow the physician to optimize individual patient management, including nutritional treatment, with the aim of preventing or reducing feeding intolerance, preventing disease progression and complications, reducing hospitalization stays, and reducing the economic burden associated with acute pancreatitis. Further clinical studies are needed to determine the perfect feeding intolerance predictor and possibilities of oral feeding improvement.

## Figures and Tables

**Table 1:** Possible risk factors of oral and enteral feeding intolerance in acute pancreatitis.

Possible Risk Factors of Oral Feeding Intolerance:	Possible Risk Factors of Enteral Feeding Intolerance:
- Lipase activity more than 2.5× above the upper limit of normal before the initiation of feeding.	- Organ failure (respiratory failure).
- Presence of SIRS at 48 h after admission.	- Pancreatic necrosis.
- Non-biliary etiology.	- Peripancreatic fluid collections.
- Presence of peri-pancreatic reservoirs.	- Hypertriglyceridemia.
- Presence of pleural effusion.	- Presence of SIRS.
- Higher Ranson and Balthazar scores.	- Acute gastrointestinal injury-grade III.
- Younger, male patients.	- Longer time from admission to commencement of enteral nutrition.
- Nicotine and alcohol abuse.	- Pancreatic infection.

**Table 2:** GCSI—Gastroparesis Cardinal Symptom Index.

	Severity of Symptoms	None	Very Mild	Mild	Moderate	Severe	Very Severe
Symptoms							
nausea/vomiting							
nausea		0	1	2	3	4	5
retching		0	1	2	3	4	5
vomiting		0	1	2	3	4	5
sensation of postprandial fullness/early satiety							
stomach fullness		0	1	2	3	4	5
not able to finish a normal-sized meal		0	1	2	3	4	5
feeling excessively full after a meal		0	1	2	3	4	5
loss of appetite		0	1	2	3	4	5
bloating							
bloating		0	1	2	3	4	5
stomach or belly visibly larger		0	1	2	3	4	5

**Table 3:** The summary of the ESPEN and ACG guidelines regarding nutrition in acute pancreatitis and feeding intolerance.

	Recommendation	Recommendation Details and Suggestions in the Case of Tolerance and Intolerance of Feeding
Oral nutrition	For patients with mild disease, oral nutrition should be initiated as soon as it is tolerated by the patient, targeted within 48 h after admission.	If an oral diet is tolerated, expand to a standard diet. If an oral diet is not tolerated, start EN.
	Initial oral feeding with a low-fat, soft oral diet (ESPEN) or low-fat solid diet (ACG).	Advise a low-fat diet for patients with gallstones pancreatitis expecting cholecystectomy and patients with hypertriglyceridemia.
	Oral feeding in patients undergoing minimally invasive necrosectomy should be initiated within the first 24 h after the procedure (ESPEN).	Introduce an oral diet in patients undergoing necrosectomy (consider the clinical status of patients including hemodynamic stability, septic parameters, gastric emptying). In patients who are unable to be fed orally, start EN.
Enteral nutrition	EN is preferred over PN in patients with AP and inability to feed orally.	Consider a semi-elemental diet in a subgroup of patients with SAP with malabsorption.
	In the case of oral feeding intolerance, EN should be started within 24–72 h of admission.	
	A standard polymeric diet is recommended.	
	In predicted SAP, ESPEN recommends initiating enteral feeding within 24–72 h of admission.	
	Continuous infusion is preferred over cyclic or bolus administration (ACG).	
	The preferred route of EN administration is nasogastric tube (ESPEN).	Slow down or withhold the supply of EN if there are symptoms of gastric feeding intolerance.
		Administration of EN via a nasojejunal tube as the preferred route in the case of the following: Digestive intolerance such as pain and vomiting, in patients with AP undergoing minimally invasive necrosectomy who are unable to fed orally; In patients with severe AP and intraabdominal pressure > 15 mmHg (starting at 20 mL/h and increasing the rate based on tolerance). Stop (temporarily) EN or start PN in the case of intra-abdominal pressure > 20 mmHg or the presence of acute compartment syndrome. EN at least in small amounts should be administered in patients with SAP and an open abdomen.
Parenteral nutrition	PN should be administered in patients with AP who do not tolerate EN, who are unable to achieve the target caloric supply, or who have contraindications to EN (ESPEN). PN should be supplemented at 0.20 g/kg per day of L-glutamine in SAP (ESPEN).	
Other substances	Probiotics are not recommended in severe AP (ESPEN). Pancreatic enzymes should not be supplemented generally except in patients with obvious pancreatic insufficiency. The addition of fiber should not be applied in patients with hemodynamic instability and bowel ischemia risk and not used routinely to improve GI motility.	
Prokinetics	Lack of recommendation in AP. In an ICU setting, the use of prokinetics may be considered if there are symptoms of gastric feeding intolerance.	Consider erythromycin (usually at dosages of 100–250 mg intravenously three times a day for two to four days), metoclopramide, or a combination of metoclopramide and erythromycin. Consider possible side effects of prokinetics. Discontinue prokinetics after three days. In patients with gastric FI not solved with prokinetic agents, start EN via a nasojejunal tube.

Prepared based on the ESPEN practical guideline on clinical nutrition in acute and chronic pancreatitis 2024 [65], the American College of Gastroenterology Guidelines: Management of Acute Pancreatitis 2024 [66], and the ESPEN practical and partially revised guideline: Clinical nutrition in the intensive care unit [39]. EN—enteral nutrition; PN—parenteral nutrition; AP—acute pancreatitis; SAP—severe acute pancreatitis; ICU—intensive care unit; FI—feeding intolerance.
